# Susceptibility of field-collected *Phlebotomus argentipes* (Diptera: Psychodidae) sand flies from Bangladesh and Nepal to different insecticides

**DOI:** 10.1186/s13071-018-2913-6

**Published:** 2018-06-04

**Authors:** Rajib Chowdhury, Murari Lal Das, Vashkar Chowdhury, Lalita Roy, Shyla Faria, Jyoti Priyanka, Sakila Akter, Narayan Prosad Maheswary, Rajaul Karim Khan, Daniel Argaw, Axel Kroeger

**Affiliations:** 1 International Centre for Diarrhoea Disease Research (icddr,b), Dhaka, 1212 Bangladesh; 2 National Institute of Preventive and Social Medicine (NIPSOM), Mohakhali, Dhaka, 1212 Bangladesh; 3 BP Koirala Institute of Health Sciences, Dharan, 56700 Nepal; 4 Dhaka College, New Market, Dhaka, 1205 Bangladesh; 5 Directorate General of Health Services (DGHS), Mohakhali, Dhaka, 1212 Bangladesh; 6 World Health Organization (WHO), 1211, 27 Geneva, Switzerland; 7 Special Programme for Research and Training in Tropical Diseases, World Health Organization, 1211, 27 Geneva, Switzerland; 8 Centre for Medicine and Society/Anthropology, University of Freiburg, Freiburg, Germany

**Keywords:** Susceptibility, Visceral leishmaniasis, *Phlebotomus argentipes*, Vector control, Alpha cypermethrin, Deltamethrin

## Abstract

**Background:**

The sand fly *Phlebotomus argentipes* is the vector for visceral leishmaniasis (VL) in the Indian sub-continent. In Bangladesh since 2012, indoor residual spraying (IRS) was applied in VL endemic areas using deltamethrin. In Nepal, IRS was initiated in 1992 for VL vector control using lambda-cyhalothrin. Irrational use of insecticides may lead to vector resistance but very little information on this subject is available in both countries. The objective of this study was to generate information on the susceptibility of the vector sand fly, *P. argentipes* to insecticide, in support of the VL elimination initiative on the Indian sub-continent.

**Methods:**

Susceptibility tests were performed using WHO test kits following the standard procedures regarding alpha cypermethrin (0.05%), deltamethrin (0.05%), lambda-cyhalothrin (0.05%), permethrin (0.75%), malathion (5%) and bendiocarb (0.1%) in six upazilas (sub-districts) in Bangladesh. In Nepal, the tests were performed for two insecticides: alpha cypermethrin (0.05%) and deltamethrin (0.05%). Adult *P. argentipes* sand flies were collected in Bangladesh from six VL endemic upazilas (sub-districts) and in Nepal from three endemic districts using manual aspirators.

**Results:**

The results show that VL vectors were highly susceptible to all insecticides at 60 minutes of exposure in both countries. In Bangladesh, corrected mortality was 100% at 15 minutes as well as 30 minutes of exposure. The study sites in Nepal, however, showed some diverse results, with a mortality rate less than 90% for 15 minutes of exposure with alpha cypermethrin and deltamethrin in two districts but was above 95% after 30 minutes of exposure.

**Conclusions:**

These results suggest that the insecticides tested can still be used in the national programmes of Bangladesh and Nepal. However, insecticide rotation should be performed to mitigate the possible development of insecticide resistance. Periodic susceptibility tests should be performed by the countries to get timely alerts regarding insecticide resistance.

## Background

Visceral leishmanisis (VL) known as kala-azar in the Indian sub-continent is a deadly parasitic disease (if untreated) caused by *Leishmania donovani* Laveran & Mesnil (Kinetoplastida: Trypanosomatidae) which is transmitted by the bite of a female sand fly *Phlebotomus argentipes* Annandale & Brunneti (Diptera: Psychodidae). *Phlebotomus argentipes* is the only incriminated vector in the Southeast Asia Region. The disease is highly prevalent in the region and is considered to be anthroponotic, and the route of transmission from human to human. VL was virtually eliminated from this part of world during the malaria eradication era through massive DDT spraying for malaria vector control [1]. It has, however, reemerged since the 1970s after the relaxation of DDT spraying [1], indicating that effective vector management is crucial to control the disease. The use of DDT was banned in Bangladesh and Nepal in 1998 and 1995, respectively, due to its environmental hazard [2, 3].

VL is endemic in 45 [4] and 18 [5] districts in Bangladesh and Nepal, respectively. A memorandum of understanding (MoU) was signed by the honorable Health Ministers from Bangladesh, India and Nepal in 2005 to eliminate VL (less than one case per 10,000 people) by 2015 [6] which was extended to 2017 [7]. Five regional strategies, i.e. (i) early diagnosis and complete case management, (ii) integrated vector management and vector surveillance, (iii) effective disease surveillance through passive and active case detection; (iv) social mobilization and building partnerships, and (v) implementation and operational research, were set up to achieve the elimination target [6].

Since the signing of the MoU, substantial progress has been made in the last few years in Bangladesh and Nepal in terms of reducing VL cases by properly utilizing its regional strategies [8]. Nepal achieved the elimination target (less than one VL case per 10,000 people in all endemic districts) and has maintained it for the last two years. Bangladesh also achieved a similar target with the exception of two upazilas (sub-districts) (Fulbaria and Trishal) [8]. Integrated vector management (IVM) is one of the major components of the regional elimination strategy. The elimination initiative has so far been successful in Nepal and in most VL endemic parts of Bangladesh. However, it is important to maintain the efforts and avoid the above described re-emergence of vectors after the malaria eradication campaign. Both countries only rely on indoor residual spraying (IRS). IRS has been used in Nepal since 1992 using lambda-cyhalothrin and alpha cypermethrin alternatively every few years for kala-azar control [9] while in Bangladesh deltamethrin has been used since 2012 [4]. The first susceptibility test of VL vectors was conducted in Nepal in 1998 for different insecticides including DDT (4%), permethrin (0.25%), deltamethrin (0.025%), lambda-cyhalothrin (0.1%), malathion (5%) and bendiocarb (0.1%) showing vector mortality rates of 98, 99, 100, 100, 100 and 100%, respectively, at 60 minutes of exposure [9]. The susceptibility test for deltamethrin was done again in Nepal in 2009 showing 96–100% mortality [3]. In Bangladesh, in 2007, 100% sand fly mortality was observed using deltamethrin [4]. However, current information on the status of insecticide resistance in the sand fly population is limited. The objective of this study was to generate evidence on the current level of insecticide susceptibility in support of the VL vector control programmes in Bangladesh and Nepal.

## Methods

### Study area and period

**Fig. 1:**
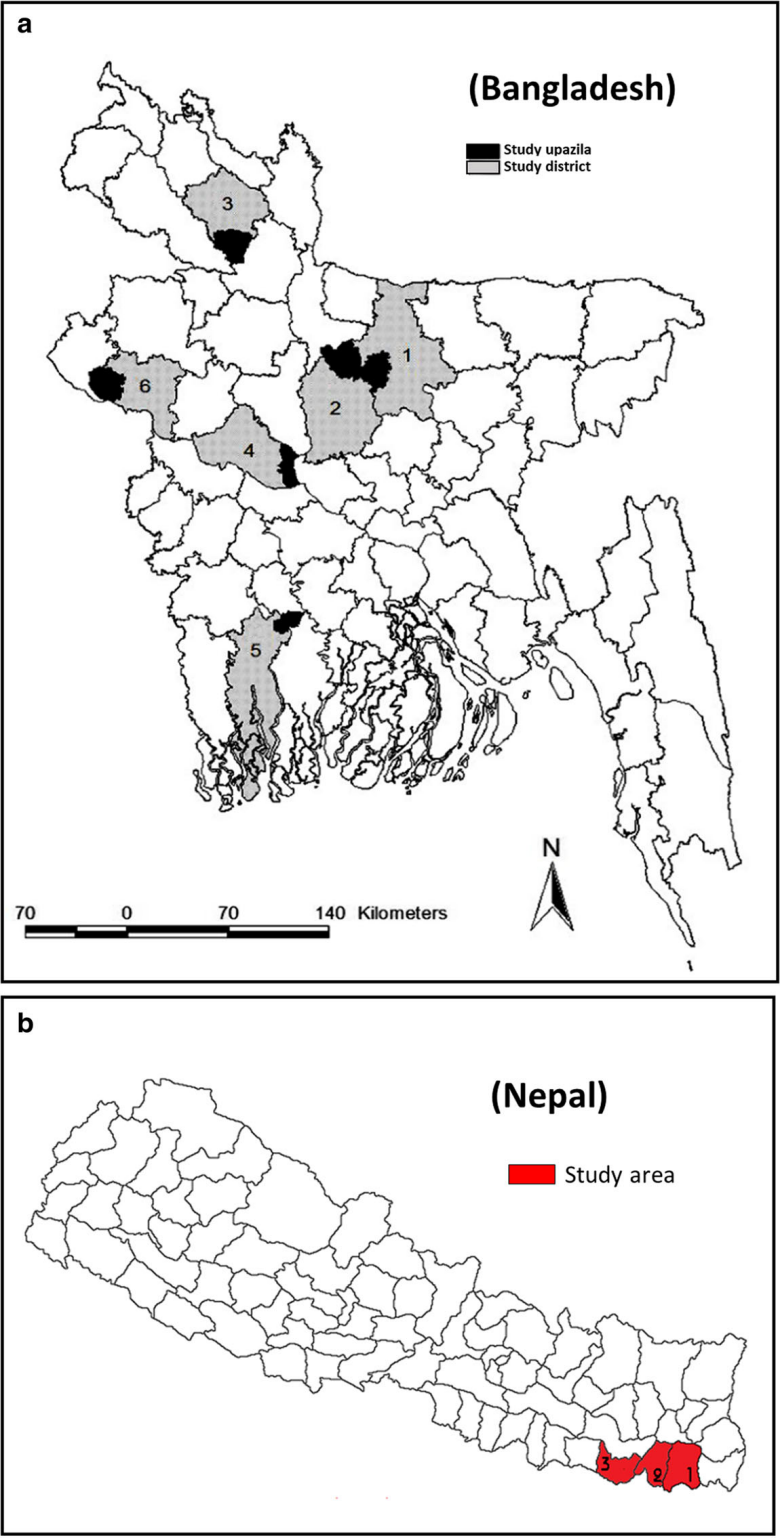
**a** Study area map of Bangladesh (*Key to upazilas*: 1, Fulbaria; 2, Madhupur; 3, Pirganj; 4, Bera; 5, Terokhada; 6, Godagari). **b** Study area map of Nepal (*Key to districts*: 1, Morang; 2, Sunssari; 3, Saptari)

The study in Bangladesh was carried out in six VL endemic upazilas (sub-districts): Fulbaria, Terokhada, Bera, Pirganj, Godagari and Madhupur under Mymensingh, Khulna, Pabna, Rangpur, Rajshahi and Tangail districts, respectively (Fig. 1a) and Bhathigachha, Tanmuna and West Pipara village development committees under Morang, Sunsari and Saptari districts, respectively, (Fig. 1b) in Nepal from October 2015 to October 2016. The study areas were selected purposively in different locations of VL endemic areas of the country. The average incidence of VL was 17.82, 2.45, 1.46, 1.14 in Fulbaria, Terokhada, Godagari and Madhupur upazilas, respectively, from 2008 to 2013. Bera, Pirganj upazilas are also endemic but the incidence is less than one case in Bangladesh [4]. Morang district had reported the highest case numbers of VL in the year 2016, in Nepal. Hence, Morang and its two neighboring districts were selected for the study. The incidence of VL was 0.46, 0.79, 0.48 and 0.51 in Morang District; 0.14, 0.17, 0.24 and 0.09 in Sunsari District and 0.75, 0.35, 0.24 and 0.15 in Saptari District, for the years 2013, 2014, 2015 and 2016, respectively [10].

### Insecticides and control paper

The tests were performed with the following insecticides in Bangladesh: alpha cypermethrin (0.05%), deltamethrin (0.05%), lambda-cyhalothrin (0.05%), permethrin (0.75%), malathion (5%) and bendiocarb (0.1%). In Nepal, the tests were performed with two insecticides: alpha cypermethrin (0.05%) and deltamethrin (0.05%). Two types of oil treated control papers were used: in the case of pyrethroids, the control papers were treated with silicone oil and in the case of organophosphate/carbamate insecticides, the control papers were treated with olive oil.

### Collection of sand flies from the field

Wild sand flies were collected from human dwellings in the above-mentioned areas in the evening from 6:30 to 20:30 h by manual aspirator and using torch light. Collected sand flies were transferred to a cage in the field laboratory where tests were performed after one hour or longer to settle down from transportation stress. Only *P. argentipes* sand flies were used for the testing after separating them from other species. A manual aspirator was used to transfer *P. argentipes* sand flies to the holding tube which was marked with a green dot. The exposure tubes were marked with a red dot and lined with insecticide-impregnated paper.

### Test procedure

The tests were performed as per the World Health Organization (WHO) recommended procedure [11, 12] with the above-mentioned insecticides. WHO test kits, insecticide-impregnated and oil treated control papers were obtained from the Universiti Sains Malaysia (WHO collaborating Center). Tests were performed at a temperature and relative humidity of 28 ± 2 °C and 70–80%, respectively. For each district, we had an independent set of bioassays with an independent control group. In Bangladesh, six replicates containing 20–25 female *P. argentipes* sand flies in each replicate were used for each insecticide and control group. In Nepal, 5 replicates were used for each insecticide together with a control group. The other procedures remained the same. After exposure, knockdown (KD) was recorded after the test period for each insecticide and control. The holding tubes were kept for recovery in dark and cool places immediately after exposure for the requisite period. Fresh sugar solution on cotton was given as supplementary food during the recovery period for 24 h. The percent mortality was calculated by counting the dead and alive *P. argentipes* sand flies after 24 h of recovery; sand fly mortality was corrected by using Abbott’s formula $\Big[\mathrm{P}=\left(\mathrm{Pi}\kern0.5em -\mathrm{C}\right)/\left(100-\mathrm{C}\right)\times 100\mathrm{P}=\frac{\mathrm{Pi}\hbox{-} \mathrm{c}}{100-\mathrm{c}}\mathrm{xd}$, where P is the corrected mortality, Pi is the percent observed mortality in immediate exposed sand flies, and C is the percent mortality in control] where control mortality should be within 5–20%. Tests were invalid in the case of control mortality exceeding 20% and those tests were repeated. The cut-off percentage was < 20% [11]. The insecticide-impregnated papers were used 4 times and after use they were stored in their respective packets. A small line was drawn in one corner of the paper to mark the number of uses for each paper.

### Sand fly identification

Female *P. argentipes* sand flies were sorted by their morphological characteristics prior to conducting the tests. All evaluated sand flies (live and dead) were identified using the standard taxonomic key described by Lewis [13, 14] and Kalra & Bang [15].

## Results

The results in Bangladesh of the susceptibility tests performed on wild populations of female *P. argentipes* sand flies with different insecticide-impregnated papers are shown in Tables 1, 2, 3, 4, 5, 6, 7, 8 and 9. The KD rate for 15, 30 and 60 minutes exposure to different insecticides varied between 19.17–40.98%, 40.48–74.19% and 81.15–96.77%, respectively. Tables 7 and 8 show that no KD was observed for any insecticides in any study sites in the control group and almost 100% of sand flies were alive 24 h post-exposure. The VL vector, *P. argentipes*, was found susceptible to all insecticides tested in the study as 100% corrected mortality was observed 24 h post-exposure (Table 9).

**Table 1:** Mortality of *P. argentipes* to alpha-cypermethrin (0.05%) in the WHO tube test (six replicates for each observation) in Bangladesh

Upazila	District	No. of sand flies exposed in 6 replicates [KD (%)]			No. of sand flies dead at 24 h (%)		
		Exposure time (min)			Exposure time (min)		
		15	30	60	15	30	60
Fulbaria	Mymensingh	120 [35 (29.16)]	122 [65 (53.28)]	125 [111 (88.80)]	120 (100)	122 (100)	125 (100)
Madhupur	Tangail	121 [32 (26.45)]	125 [59 (47.20)]	128 [115 (89.84)]	121 (100)	125 (100)	128 (100)
Pirgonj	Rangpur	122 [37 (30.33)]	120 [69 (57.50)]	122 [109 (89.34)]	122 (100)	120 (100)	122 (100)
Bera	Pabna	124 [39 (31.45)]	121 [62 (51.24)]	123 [111 (90.24)]	124 (100)	121 (100)	123 (100)
Terokhada	Khulna	123 [41 (33.33)]	124 [71 (57.26)]	126 [112 (88.89)]	123 (100)	124 (100)	126 (100)
Godagari	Rajshahi	126 [43 (34.13)]	124 [63 (50.81)]	122 [110 (90.16)]	126 (100)	124 (100)	122 (100)

*Abbreviation*: *KD* knockdown rate

**Table 2:** Mortality status of *P. argentipes* to deltamethrin (0.05%) in the WHO tube test (six replicates for each observation) in Bangladesh

Upazila	District	No. of sand flies exposed in 6 replicates [KD (%)]			No. of sand flies dead at 24 h (%)		
		Exposure time (min)			Exposure time (min)		
		15	30	60	15	30	60
Fulbaria	Mymensingh	120 [28 (23.33)]	121 [53 (43.80) ]	120 [105 (87.5)]	120 (100)	121 (100)	120 (100)
Madhupur	Tangail	124 [32 (25.81)]	121 [55 (45.45)]	121 [107 (88.43)]	124 (100)	121 (100)	121 (100)
Pirgonj	Rangpur	120 [35 (29.17)]	126 [63 (50.00)]	120 [109 (90.83)]	120 (100)	126 (100)	120 (100)
Bera	Pabna	126 [35 (27.78)]	122 [59 (48.36)]	120 [105 (87.5)]	126 (100)	122 (100)	120 (100)
Terokhada	Khulna	121 [42 (34.71)]	122 [66 (54.10)]	126 [112 (88.89)]	121 (100)	122 (100)	126 (100)
Godagari	Rajshahi	124 [39 (31.45)]	123 [57 (46.34)]	127 [114 (89.76)]	124 (100)	123 (100)	127 (100)

*Abbreviation*: *KD* knockdown rate

**Table 3:** Mortality status of *P. argentipes* to lambda-cyhalothrin (0.05%) in the WHO tube test (six replicates for each observation) in Bangladesh

Upazila	District	No. of sand fly exposed in 6 replicates [KD (%)]			No. of sand flies dead at 24 h (%)		
		Exposure time (min)			Exposure time (min)		
		15	30	60	15	30	60
Fulbaria	Mymensingh	120 [23 (19.17)]	122 [51 (41.80)]	120 [108 (90.00)]	120 (100)	122 (100)	120 (100)
Madhupur	Tangail	126 [28 (22.22)]	123 [52 (42.28)]	122 [112 (91.80)]	126 (100)	123 (100)	122 (100)
Pirgonj	Rangpur	126 [35 (27.78)]	121 [55 (45.45)]	124 [112 (90.32)]	126 (100)	121 (100)	124 (100)
Bera	Pabna	122 [31 (25.41)]	124 [59 (47.58)]	120 [110 (91.67)]	122 (100)	124 (100)	120 (100)
Terokhada	Khulna	121 [29 (24.97)]	120 [51 (42.50)]	122 [108 (88.52)]	121 (100)	120 (100)	122 (100)
Godagari	Rajshahi	123 [24 (19.51)]	126 [51 (40.48)]	126 [110 (87.30)]	123 (100)	126 (100)	126 (100)

*Abbreviation*: *KD* knockdown rate

**Table 4:** Mortality status of *P. argentipes* to permethrin (0.75%) in the WHO tube test (six replicates for each observation) in Bangladesh

Upazila	District	No. of sand flies exposed in 6 replicates [KD (%)]			No. of sand flies dead at 24 h (%)		
		Exposure time (min)			Exposure time (min)		
		15	30	60	15	30	60
Fulbaria	Mymensingh	121 [26 (21.49)]	123 [64 (52.03)]	122 [108 (88.52)]	121 (100)	123 (100)	122 (100)
Madhupur	Tangail	126 [28 (22.22)]	125 [65 (52.00)]	120 [103 (85.83)]	126 (100)	125 (100)	120 (100)
Pirgonj	Rangpur	125 [30 (24.00)]	122 [62 (50.82)]	120 [105 (87.5)]	125 (100)	122 (100)	120 (100)
Bera	Pabna	121 [31 (25.61)]	124 [57 (45.97)]	122 [108 (88.52)]	121 (100)	124 (100)	122 (100)
Terokhada	Khulna	122 [28 (22.95)]	121 [58 (47.93)]	124 [105 (84.68)]	122 (100)	121 (100)	124 (100)
Godagari	Rajshahi	123 [30 (24.39)]	120 [63 (52.50)]	120 [106 (88.33)]	123 (100)	120 (100)	120 (100)

*Abbreviation*: *KD* knockdown rate

**Table 5:** Mortality status of *P. argentipes* to malathion (5%) in the WHO tube test (six replicates for each observation) in Bangladesh

Upazila	District	No. of sand flies exposed in 6 replicates [KD (%)]			No. of sand flies dead at 24 h (%)		
		Exposure time (min)			Exposure time (min)		
		15	30	60	15	30	60
Fulbaria	Mymensingh	122 [53 (43.44)]	124 [92 (74.19)]	124 [120 (96.77)]	122 (100)	124 (100)	124 (100)
Madhupur	Tangail	122 [50 (40.98)]	120 [87 (72.50)]	120 [112 (93.33)]	122 (100)	120 (100)	120 (100)
Pirgonj	Rangpur	120 [53 (44.17)]	120 [83 (69.17)]	121 [114 (94.21)]	120 (100)	120 (100)	121 (100)
Bera	Pabna	126 [57 (45.24)]	122 [86 (70.49)]	123 [111 (90.24)]	126 (100)	122 (100)	123 (100)
Terokhada	Khulna	125 [55 (44.00)]	126 [91 (72.22)]	122 [115 (94.26)]	125 (100)	126 (100)	122 (100)
Godagari	Rajshahi	121 [54 (44.63)]	126 [89 (70.63)]	124 [116 (93.55)]	121 (100)	126 (100)	124 (100)

*Abbreviation*: *KD* knockdown rate

**Table 6:** Mortality status of *P. argentipes* to bendiocarb (0.1%) in the WHO tube test (six replicates for each observation) in Bangladesh

Upazila	District	No. of sand flies exposed in 6 replicates [KD (%)]			No. of sand flies dead at 24 h (%)		
		Exposure time (min)			Exposure time (min)		
		15	30	60	15	30	60
Fulbaria	Mymensingh	122 [42 (35.00)]	125 [81 (66.39)]	123 [102 (82.93)]	122 (100)	125 (100)	123 (100)
Madhupur	Tangail	120 [48 (39.34)]	122 [82 (67.21)]	122 [99 (81.15)]	118 (98.33)	121 (99.18)	122 (100)
Pirgonj	Rangpur	122 [47 (38.52)]	122 [79 (64.75)]	120 [98 (81.67)]	121 (99.18)	121 (99.18)	120 (100)
Bera	Pabna	126 [52 (41.27)]	120 [82 (67.21)]	123[104 (84.55)]	125 (99.21)	119 (99.17)	123 (100)
Terokhada	Khulna	124 [51 (41.13)]	120 [83 (68.03)]	123[101 (82.11)]	122 (98.39)	120 (100)	123 (100)
Godagari	Rajshahi	125 [44 (35.20)]	126 [85 (69.67)]	120 [101 (84.17)]	123 (98.4)	124 (98.41)	120 (100)

*Abbreviation*: *KD* knockdown rate

**Table 7:** Control mortality in silicon oil: PY status of *P. argentipes* in the WHO tube test (six replicates for each observation) in Bangladesh

Upazila	District	No. of sand flies exposed in 6 replicates [KD (%)]			No. of sand flies dead at 24 h (%)		
		Exposure time (min)			Exposure time (min)		
		15	30	60	15	30	60
Fulbaria	Mymensingh	120 [0 (0)]	124 [0 (0)]	126 [0 (0)]	0	0	7 (5.56)
Madhupur	Tangail	122 [0 (0)]	120 [0 (0)]	120 [0 (0)]	0	8 (6.67)	0
Pirgonj	Rangpur	125 [0 (0)]	120 [0 (0)]	120 [0 (0)]	0	0	0
Bera	Pabna	121 [0 (0)]	123 [0 (0)]	120 [0 (0)]	0	0	0
Terokhada	Khulna	123 [0 (0)]	122 [0 (0)]	123 [0 (0)]	0	0	9 (7.32)
Godagari	Rajshahi	124 [0 (0)]	120 [0 (0)]	123 [0 (0)]	0	9 (7.5)	0

*Abbreviations*: *KD* knockdown rate, *PY* pyrethroid

**Table 8:** Control mortality in olive oil: OP/CR status of *P. argentipes* in the WHO tube test (six replicates for each observation) in Bangladesh

Upazila	District	No. of sand flies exposed in 6 replicates [KD (%)]			No. of sand flies dead at 24 h (%)		
		Exposure time (min)			Exposure time (min)		
		15	30	60	15	30	60
Fulbaria	Mymensingh	121 [0 (0)]	124 [0 (0)]	126 [0 (0)]	0	8 (6.45)	0
Madhupur	Tangail	125 [0 (0)]	120 [0 (0)]	120 [0 (0)]	0	0	9 (7.5)
Pirgonj	Rangpur	120 [0 (0)]	122 [0 (0)]	120 [0 (0)]	0	0	8 (6.67)
Bera	Pabna	120 [0 (0)]	123 [0 (0)]	120 [0 (0)]	0	0	0
Terokhada	Khulna	124 [0 (0)]	120 [0 (0)]	122 [0 (0)]	0	0	0
Godagari	Rajshahi	123 [0 (0)]	120 [0 (0)]	121 [0 (0)]	0	0	0

*Abbreviations*: *KD* knockdown rate, *OP* organophosphates, *CR* carbamate

**Table 9:** Susceptibility status of *P. argentipes* to different insecticides in the WHO tube test (six replicates for each observation) for 15, 30 and 60 minutes exposure in Bangladesh. Values without parentheses indicate observation at 15 minutes, those in parentheses - at 30 minutes and those in square brackets - at 60 minutes

Insecticide	District	Mymensingh	Tangail	Rangpur	Pabna	Khulna	Rajshahi
	Upazila	Fulbaria	Madhupur	Pirganj	Bera	Terokhada	Godagari
Alpha-cypermethrin (0.05%)	No. of exposed	120 (122) [125]	121 (125) [128]	122 (120) [122]	124 (121) [123]	123 (124) [126]	126 (124) [122]
	% mortality	100 (100) [100]	100 (100) [100]	100 (100) [100]	100 (100) [100]	100 (100) [100]	100 (100) [100]
	% control mortality	0 (0) [5.56]	0 (6.67) [0]	0 (0) [0]	0 (0) [0]	0 (0) [7.32]	0 (7.5) [0]
	Corrected mortality	100 (100) [100]	100 (100) [100]	100 (100) [100]	100 (100) [100]	100 (100) [100]	100 (100) [100]
	Status	S	S	S	S	S	S
Deltamethrin (0.05%)	No. of exposed	120 (121) [120]	124 (121) [121]	120 (126) [120]	126 (122) [120]	121 (122) [126]	124 (123) [127]
	% mortality	100 (100) [100]	100 (100) [100]	100 (100) [100]	100 (100) [100]	100 (100) [100]	100 (100) [100]
	% control mortality	0 (0) [5.56]	0 (6.67) [0]	0 (0) [0]	0 (0) [0]	0 (0) [7.32]	0 (7.5) [0]
	Corrected mortality	100 (100) [100]	100 (100) [100]	100 (100) [100]	100 (100) [100]	100 (100) [100]	100 (100) [100]
	Status	S	S	S	S	S	S
Lambda-cyhalothrin (0.05%)	No. of exposed	120 (122) [120]	126 (123) [122]	126 (121) [124]	122 (124) [120]	121 (120) [122]	123 (126) [126]
	% mortality	100 (100) [100]	100 (100) [100]	100 (100) [100]	100 (100) [100]	100 (100) [100]	100 (100) [100]
	% control mortality	0 (0) [5.56]	0 (6.67) [0]	0 (0) [0]	0 (0) [0]	0 (0) [7.32]	0 (7.5) [0]
	Corrected mortality	100 (100) [100]	100 (100) [100]	100 (100) [100]	100 (100) [100]	100 (100) [100]	100 (100) [100]
	Status	S	S	S	S	S	S
Permethrin (0.75%)	No. of exposed	121(123) [122]	126 (125) [120]	125 (122) [120]	121 (124) [122]	122 (121) [124]	123 (120) [120]
	% mortality	100 (100) [100]	100 (100) [100]	100 (100) [100]	100 (100) [100]	100 (100) [100]	100 (100) [100]
	% control mortality	0 (0) [5.56]	0 (6.67) [0]	0 (0) [0]	0 (0) [0]	0 (0) [7.32]	0 (7.5) [0]
	Corrected mortality	100 (100) [100]	100 (100) [100]	100 (100) [100]	100 (100) [100]	100 (100) [100]	100 (100) [100]
	Status	S	S	S	S	S	S
Malathion (5%)	No. of exposed	122 (124) [124]	122 (120) [120]	120 (120) [121]	126 (122) [123]	125 (126) [122]	121 (126) [124]
	% mortality	100 (100) [100]	100 (100) [100]	100 (100) [100]	100 (100) [100]	100 (100) [100]	100 (100) [100]
	% control mortality	0 (6.45) [0]	0 (0) [7.5]	0 (0) [6.67]	0 (0) [0]	0 (0) [0]	0 (0) [0]
	Corrected mortality	100 (100) [100]	100 (100) [100]	100 (100) [100]	100 (100) [100]	100 (100) [100]	100 (100) [100]
	Status	S	S	S	S	S	S
Bendiocarb (0.1%)	No. of exposed	120 (122) [123]	122 (125) [122]	122 (122) [120]	126 (120) [123]	124 (120) [123]	125 (126) [120]
	% mortality	100 (100) [100]	100 (100) [100]	100 (100) [100]	100 (100) [100]	100 (100) [100]	100 (100) [100]
	% control mortality	0 (6.45) [0]	0 (0) [7.5]	0 (0) [6.67]	0 (0) [0]	0 (0) [0]	0 (0) [0]
	Corrected mortality	100 (100) [100]	100 (100) [100]	100 (100) [100]	100 (100) [100]	100 (100) [100]	100 (100) [100]
	Status	S	S	S	S	S	S

*Abbreviation*: *S* susceptible

The study results in Nepal are shown in Tables 10, 11, 12, 13 and 14. They show that the KD rates of alpha cypermethrin and deltamethrin were 16.22–27.27%, 77.78–83.18% and 93.20–97.27% for 15, 30 and 60 minutes exposure, respectively. The mortality rate was 100 % for 60 minutes of exposure for both insecticides in all three study districts but different results were found for 15 and 30 minutes of exposure (Table 14). These results indicate the need for establishing the susceptibility status of vector sand flies for above tested (alpha-cypermethrin and deltamethrin) as well as other insecticides in the pipeline for IRS in Nepal.

**Table 10:** Mortality status of *P. argentipes* to alpha-cypermethrin (0.05%) in the WHO tube test (five replicates for each observation) in Nepal

District	Village Development Committee	No. of sand flies exposed in 5 replicates [KD (%)]			No. of sand flies dead at 24 h (%)		
		Exposure time (min)			Exposure time (min)		
		15	30	60	15	30	60
Morang	Bhathigachh	111 [23 (20.72)]	108 [84 (77.78)]	109 [106 (97.25)]	100 (89.89)	108 (100)	109 (100)
Sunsari	Tanmuna	110 [30 (27.27)]	107 [89 (83.18)]	106 [100 (94.34)]	105 (95.45)	107 (100)	106 (100)
Saptari	West Pipara	111 [18 (16.22)]	105 [82 (78.10)]	104 [99 (95.19)]	106 (95.10)	105 (100)	104 (100)

*Abbreviation*: *KD* knockdown rate

**Table 11:** Mortality status of *P. argentipes* to deltamethrin (0.05%) in the WHO tube test (five replicates for each observation) in Nepal

District	Village Development Committee	No. of sand flies exposed in 5 replicates [KD (%)]			No. of sand flies dead at 24 h (%)		
		Exposure time (min)			Exposure time (min)		
		15	30	60	15	30	60
Morang	Bhathigachh	114 [31 (27.19)]	103 [75 (72.82)]	110 [107 (97.27)]	107 (93.86)	99 (95.73)	110 (100)
Sunsari	Tanmuna	104 [21 (20.19)]	108 [88 (81.48)]	103 [96 (93.20)]	93 (89.42)	103 (95.37)	103 (100)
Saptari	West Pipara	114 [19 (16.67)]	108 [79 (73.15)]	109 [103 (94.50)]	109 (95.48)	108 (100)	109 (100)

*Abbreviation*: *KD* knockdown rate

**Table 12:** Control mortality in silicon oil: PY status of *P. argentipes* in the WHO tube test (five replicates for each observation) for alpha-cypermethrin in Nepal

District	Village Development Committee	No. of sand flies exposed in 5 replicates [KD (%)]			No. of sand flies dead at 24 h (%)		
		Exposure time (min)			Exposure time (min)		
		15	30	60	15	30	60
Morang	Bhathigachh	102 [0 (0)]	104 [0 (0)]	117 [0 (0)]	2 (1.96)	2 (1.92)	6 (5.13)
Sunsari	Tanmuna	104 [0 (0)]	104 [0 (0)]	107 [0 (0)]	0	0	0
Saptari	West Pipara	112 [0 (0)]	103 [0 (0)]	115 [0 (0)]	9 (8.04)	2 (1.94)	10 (8.70)

*Abbreviations*: *KD* knockdown rate, *PY* pyrethroid

**Table 13:** Control mortality in silicon oil: PY status of *P. argentipes* in the WHO tube test (five replicates for each observation) for deltamethrin in Nepal

District	Village Development Committee	No. of sand flies exposed in 5 replicates [KD (%)]			No. of sand flies dead at 24 h (%)		
		Observation time (min)			Observation time (min)		
		15	30	60	15	30	60
Morang	Bhathigachh	102 [0 (0)]	110 [0 (0)]	120 [0 (0)]	0	10 (9.09)	0
Sunsari	Tanmuna	104 [0 (0)]	100 [0 (0)]	110 [0 (0)]	0	0	0
Saptari	West Pipara	104 [0 (0)]	104 [0 (0)]	110 [0 (0)]	3 (2.88)	4 (3.85)	5 (4.55)

*Abbreviations*: *KD* knockdown rate, *PY* pyrethroid

**Table 14:** Susceptibility status of *P. argentipes* to different insecticides in the WHO tube test (six replicates for each observation) for 15, 30 and 60 minutes exposure in Nepal. Values without parentheses indicate observation at 15 minutes, those in parentheses - at 30 minutes and those in square brackets - at 60 minutes

Insecticide	District	Morang	Sunsari	Saptari
	Village development committee	Bhathigachh	Tanmuna	West Pipara
Alpha-cypermethrin (0.05%)	No. exposed	111 (108) [109]	110 (107) [106]	111 (105) [104]
	% mortality	90.09 (100) [100]	95.45 (100) [100]	95.50 (100) [100]
	% control mortality	1.96 (1.92) [5.13]	0 (0) [0]	8.04 (1.94) [8.70]
	Corrected mortality	89.89 (100) [100]	95.45 (100) [100]	95.10 (100) [100]
	Status	S?	S	S
Deltamethrin (0.05%)	No. exposed	114 (103) [110]	104 (108) [103]	114 (108) [109]
	% mortality	93.86 (96.12) [100]	89.42 (95.37) [100]	95.61 (100) [100]
	% control mortality	0 (9.09) [0]	0 (0) [0]	2.88 (3.85) [4.55]
	Corrected mortality	93.86 (95.73) [100]	89.42 (95.37) [100]	95.48 (100) [100]
	Status	S	S?	S

*Abbreviations*: *S* susceptible

**Fig. 2:**
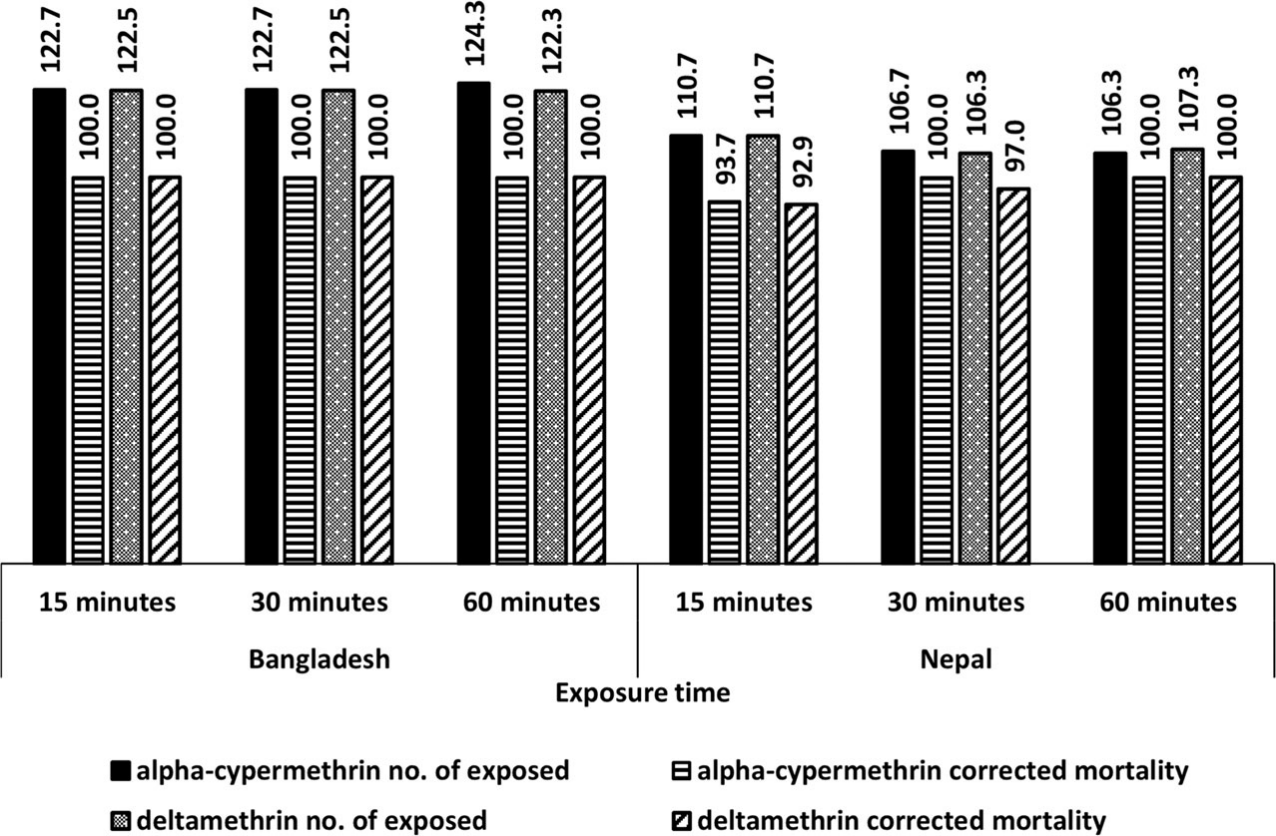
Average number of *Phlebotomus argentipes* exposed and their corrected mortality for two common insecticides, alpha-cypermethrin and deltamethrin tested in each study sites in Bangladesh (six study sites and six replicates in each site) and Nepal (three study sites and five replicates in each site)

In this study, two insecticides, alpha-cypermethrin and deltamethrin, were used in both Bangladesh and Nepal; the tests performed, and the comparative findings are shown in Fig. 2. In Bangladesh, the mortality was 100% for both insecticides for 15, 30 and 60 minutes exposure, whilst this was the case only for 30 minutes exposure for alpha-cypermethrin and 60 minutes exposure for both insecticides in Nepal. In Nepal, the mortality dropped to 92.9 and 93.7 from 100% for 15 minutes exposure for both insecticides and to 97.0 from 100% at 30 minutes exposure for deltamethrin.

## Discussion

Insect-borne diseases remain a major public health concern in resource constrained situations. About 30 different species of phlebotomine sand flies are responsible for transmitting leishmaniasis worldwide [16]. Vector control is an important element to minimize the vector-borne disease burden in the countries and in fact, it heavily relies upon different insecticides. The inappropriate application (overuse and misuse) of insecticides have led to the emergence of resistance, which undermines the potency of vector control. Usually there are four possible types of insecticides resistance observed, i.e. (i) increased metabolism to non-toxic products, (ii) decreased target site sensitivity, (iii) decreased rates of insecticide penetration and (iv) increased rates of insecticide excretion, for four major classes of insecticides (organochlorines, organophosphates, carbamates and pyrethroids) [17]. Development of resistance is an evolutionary phenomenon caused by either behavioural avoidance or physiological or biochemical factors of any targeted insects. This can be tackled judiciously by implementing appropriate and comprehensive resistance monitoring and management strategies within the framework of integrated vector management. Insecticide resistance occurs when the insecticide no longer binds to its target or through detoxification enzyme-based resistance, which occurs when enhanced levels or modified activities of esterases, oxidases, or glutathione S-transferases prevent the insecticide from reaching its site of action [18].

In the present study, we did not perform assays of the susceptibility of sand flies because the lethal doses of all classes of insecticides are generally similar to those for mosquitoes [19] and determined the doses for the purpose of discriminating concentrations in routine insecticide resistance monitoring. This is now well established and widely adopted for the purposes of testing and monitoring insecticide resistance in mosquitoes and other disease vectors [20]. Wild-caught *P. argentipes* female sand flies were used in the present study as none of the study countries have a sand fly colony.

The present study confirmed that *P. argentipes* sand flies in Bangladesh are susceptible to alpha-cypermethrin, deltamethrin, lambda-cyhalothrin, permethrin, malathion and bendiocarb as 100% mortality was observed at 60 minutes of exposure. In Nepal, incipient signs of resistance development were observed: the mortality rate was below 90% after 15 minutes and about 95% after 30 minutes of exposure to pyrethroid insecticides but 100% after 60 minutes of exposure for both insecticides which is slightly different (the mortality was between 96–99% for 60 minutes for deltamethrin 0.05%) from an earlier study conducted in Nepal and India [3]. Two insecticides (deltamethrin and alpha-cypermethrin) were tested in the both countries and the findings indicate that the *P. argentipes* populations are on the way to developing tolerance against both insecticides in Nepal whereas they are still 100% susceptible in Bangladesh. In Nepal, pyrethroid based (alpha-cypermethrin, lambda-cyhalothrin and deltamethrin) IRS has been in practice for a long time and various types of pyrethroids are in use in different years. Rotation of insecticide for IRS is still not foreseen in Nepal, hence it is essential to monitor the available insecticide for its efficacy to control vectors of VL. It is also worth mentioning that if the potential emergence of resistance is observed, we need to go for next step (molecular mechanism) of confirmation resistance test.

The VL vector control programmes in Bangladesh and Nepal are completely dependent on insecticide-based interventions, mainly IRS using synthetic pyrethroids (deltamethrin, alpha-cypermethrin and lambda-cyhalothrin) in human dwellings as well as in cattle sheds. Nepal reached the elimination target (less than one case per 10,000 people at district level) for the last few years; Bangladesh has yet to achieve it as a few upazilas (sub-districts) are still reporting incidence rates above the elimination target. Continuous vector control activities with rational use of quality insecticide are essential to sustain this achievement and eliminate the disease. As mentioned before, VL was virtually eliminated from Bangladesh and Nepal due to the massive use of DDT for IRS during the malaria eradication era [1, 2] but reappeared in the sub-continent due to a lack of effective vector control activities in the post-eradication phase. IRS is an expensive operation so it must be carried out using appropriate insecticides to achieve the optimum results against the targeted vector. Periodic monitoring insecticide efficacy through susceptibility tests (by WHOPES suggested method) is needed to have updated information on vector susceptibility to insecticides. IRS is an operationally challenging activity in terms of maintaining its optimum quality. Due to poor operation and relatively lower disease burden, the community acceptance of IRS is gradually decreasing in the Indian sub-continent (personal observation of RC). A study in India and Nepal identified that IRS is effective on vector reduction when it was carried out by the research team in a controlled situation but it was deficient when it was carried out by the National Programme [21]. Long-term irrational use of insecticide may develop tolerance or resistance on its targeted insects which we observed in Nepal. The WHOPES-recommended susceptibility test kits and insecticide-impregnated paper are only available at Universiti Sains Malaysia and to obtain them in time is difficult which affects the planned activity so WHO may come forward to find out the solution.

In the case of Bangladesh, the chance of cross-resistance is very unlikely as there were virtually no vector control activities for a long time (1997–2012) [4, 22]. Limited scale IRS was conducted in 2012 using deltamethrin 5WP in Bangladesh and since then it has continued, usually with two rounds of spraying. While IRS has been carried out in Nepal using synthetic pyrethroids for a long time as part of VL vector control activities, the current study did not evaluate the cross-resistance as a fully susceptible reference strain of *P. argentipes* was not available.

## Conclusions

In conclusion, it is recommended that the national control programme in Bangladesh and Nepal can use alpha-cypermethrin, deltamethrin, lambda-cyhalothrin, permethrin, malathion and bendiocarb for VL vector control. In Nepal, however, deltamethrin or alpha-cypermethrin should be used judiciously. In both cases, insecticide rotation should be performed to mitigate the possible development of insecticide resistance. Periodic susceptibility tests should be performed by the countries to get timely alerts regarding insecticide resistance.

## Abbreviations

BMRC: Bangladesh Medical Research Council
BPKIHS: BP Koirala Institute of Health Sciences
DDT: Dichlorodiphenyltrichloroethane
IRS: Indoor residual spraying
IVM: Integrated vector management
KD: Knockdown
MoU: Memorandum of understanding
OP/CR: Organophosphate/carbamate
PY: Pyrethroid
VL: Visceral leishmanisis
WHO: World Health Organization
WHOPES: World Health Organization Pesticide Evaluation Scheme
WP: Wettable powder
